# Prophylaxis Strategies for SARS‐CoV‐2 Infection in Adults With Hematological Malignancies in Saudi Arabia: Consensus Statements From the Working Group on COVID‐19 Management in Hematological Malignancies

**DOI:** 10.1002/cnr2.70730

**Published:** 2026-10-26

**Authors:** Mubarak Al‐Mansour, Ayman Alhejazi, Manal Ahmed Alsaggaf, Hafiz Mosa Malhan, Riad El Fakih, Reyad Dada, Ghazi Saud Alotaibi, Reem S. Almaghrabi, Majid Alshamrani

**Affiliations:** ^1^ Department of Medical Education, College of Medicine King Saud Bin Abdul Aziz University for Health Sciences (KSAU‐HS), King Abdullah International Medical Research Centre (KAIMRC), Ministry of National Guard Health Affairs (MNGHA), King Abdulaziz Medical City Jeddah Saudi Arabia; ^2^ Adult Medical Oncology Princess Noorah Oncology Centre, King Abdullah International Medical Research Centre (KAIMRC), Ministry of National Guard Health Affairs (MNGHA), King Abdulaziz Medical City Jeddah Saudi Arabia; ^3^ Division of Adult Haematology and Hematopoietic Stem Cell Transplantation, Department of Oncology King Abdulaziz Medical City Riyadh Saudi Arabia; ^4^ Saudi German Hospital Makkah Saudi Arabia; ^5^ Prince Mohmmad bin Nasser Hospital Jazan Saudi Arabia; ^6^ King Faisal Specialist Hospital and Research Centre Riyadh Saudi Arabia; ^7^ Department of Oncology King Faisal Specialist Hospital and Research Centre Jeddah Saudi Arabia; ^8^ Department of Medicine King Saud University Riyadh Saudi Arabia; ^9^ Transplant Infectious Diseases, Organ Transplant Centre of Excellence King Faisal Specialist Hospital and Research Centre Riyadh Saudi Arabia; ^10^ Infection Prevention and Control Program King Abdulaziz Medical City, King Saud bin Abdulaziz University for Health Sciences, King Abdullah International Medical Research Centre, Ministry of National Guard Health Affairs Riyadh Saudi Arabia

**Keywords:** COVID‐19, hematological malignancies, monoclonal antibodies, vaccination

## Abstract

**Background:**

Despite the end of the pandemic phase, patients with hematological malignancies (HMs) remain vulnerable to severe COVID‐19 infection and mortality. Prophylaxis strategies, including vaccination and monoclonal antibodies (mAbs) like AZD7442, are crucial for protection. In Saudi Arabia, the incidence of HMs has risen, emphasizing the need for locally adapted prophylaxis strategies.

**Aims:**

This consensus provides recommendations for severe acute respiratory syndrome coronavirus 2 (SARS‐CoV‐2) infection prophylaxis in adults with HMs in Saudi Arabia.

**Methods:**

We employed a modified Delphi approach to develop recommendations for minimizing the risk of SARS‐CoV‐2 infection in patients with HMs. A multidisciplinary expert panel of hematologists, oncologists, and infectious disease specialists was formed. The Delphi process involved three voting rounds. Consensus was achieved if there was ≥ 80% agreement.

**Results:**

The present consensus consists of 24 statements. The panel agreed that the currently recommended COVID‐19 vaccination is the frontline prophylactic strategy for SARS‐CoV‐2 prophylaxis or severe COVID‐19 in patients with HMs. They also agreed that patients with HMs should receive at least one booster dose of an updated COVID‐19 vaccine, and that vaccination should not delay scheduled treatment. With regard to patients in the high‐risk group, pre‐exposure long‐acting mAbs may be considered when activity against currently circulating SARS‐CoV‐2 variants has been demonstrated, and the agent is locally available and approved by the relevant regulatory authorities. The mAbs should be started, even if the patient is under treatment. In the case of vaccinated patients, the long‐acting mAbs should be administered at least 2 weeks after vaccination.

**Conclusion:**

There is a need for tailored prophylactic strategies to protect high‐risk immunocompromised patients from COVID‐19. Key recommendations include the need for booster doses of the updated vaccines, according to national availability, and the use of long‐acting mAbs for pre‐exposure prophylaxis, only when activity against circulating variants has been demonstrated, and the product is locally available and approved by the relevant regulatory authority.

## 1 Background

The emergence of the COVID‐19 pandemic has been associated with a substantial increase in morbidity and fatalities among various vulnerable populations. Among these groups, patients with hematological malignancies (HMs) have emerged as a particularly vulnerable cohort, with a higher risk of severe infection, complications, hospitalization, intensive care unit (ICU) admission, and mortality compared to the general population [1, 2]. Patients with HMs often experience immunosuppression due to the underlying disease and its associated treatments, such as chemotherapy, radiotherapy, stem cell transplantation, and immunomodulatory therapies. This immunocompromised state renders them more susceptible to severe acute respiratory syndrome coronavirus 2 (SARS‐CoV‐2) infections and predisposes them to severe COVID‐19 manifestations, prolonged viral shedding, and poor clinical outcomes [2, 3, 4]. Although we are currently in the endemic phase of COVID‐19, characterized by less mortality and severity in the general population, patients with HMs still face an increased risk of severe COVID‐19 and overall mortality [5].

Prophylaxis strategies for SARS‐CoV‐2 infection in adults with HMs encompass a multifaceted approach, including vaccination, pharmacological interventions, and non‐pharmacological measures. Although vaccinations remain the most important prophylactic measure, patients with HMs typically show suboptimal responses to vaccination [6]. Additionally, the waning of immune responses has been documented alongside breakthrough infections [7], leading to the recommendations of booster immunization with bivalent vaccines [2]. In light of these limitations, mAbs can play a significant role in SARS‐CoV‐2 infection prophylaxis among patients with HMs [8]. Adjunct AZD7442 is a combination of two long‐acting mAbs, tixagevimab and cilgavimab (T/C), that aim to provide extended protection against COVID‐19 in high‐risk populations, such as patients with HMs. In the PROVENT Phase 3 trial, T/C reduced the risk of symptomatic COVID‐19 by 77% in high‐risk individuals who had not been previously infected with SARS‐CoV‐2 or vaccinated against COVID‐19 [9]. These findings provide historical proof of concept for antibody‐based pre‐exposure prophylaxis but should not be interpreted as evidence of effectiveness against subsequently emerging variants. Other long‐acting mAbs, such as VYD222 [10], REGN17092 [11], and AER‐800 [12], are currently under investigation. Contemporary prophylactic use of any anti‐SARS‐CoV‐2 mAbs should be considered only when activity against circulating variants has been demonstrated, and the product is locally approved and available.

The TACKLE Phase 3 trial evaluated T/C for the treatment of non‐hospitalized patients with mild to moderate COVID‐19 and demonstrated a reduction in the risk of severe COVID‐19 or death [13].

The incidence of HMs has notably increased in Saudi Arabia [14]. Previous epidemiological studies reported that leukemia is the most common cancer type among males in Saudi Arabia, accounting for 8% of all male cancer cases in 2020 [14]. Likewise, non‐Hodgkin's lymphoma (NHL) and Hodgkin's lymphoma have become the third and sixth most common malignancies among Saudi males, respectively, with lymphoma cases accounting for nearly 12.4% of all new cancer cases in 2020 [15]. Recent data have also shown an almost 100% increase in the incidence of Hodgkin's lymphoma from 2001 to 2020 [16]. Given the significant burden of HMs in Saudi Arabia, it is crucial to develop effective prophylaxis strategies to protect this vulnerable population from SARS‐CoV‐2 infection and its associated complications.

The present consensus provides locally adopted recommendations for prophylaxis strategies for SARS‐CoV‐2 infection in adults with HMs in Saudi Arabia.

## 2 Methods

### 2.1 Study Design and Panel Recruitment

The Working Group on COVID‐19 Management in HMs, comprising a multidisciplinary team of experts in hematology, oncology, and infectious diseases, has collaboratively developed these recommendations to minimize infection risk and improve clinical outcomes in this high‐risk group. We adopted a three‐step Delphi method, which involved a series of iterative rounds of questionnaires, followed by controlled feedback and statistical analysis, to converge the opinions of the panel [17].

Potential panelists were identified using non‐probability purposive sampling. Eligibility was based on clinical expertise in hematology, hematopoietic stem‐cell transplantation (HSCT), oncology, infectious diseases, or transplant infectious diseases; an active research or publication profile relevant to hematological malignancies or COVID‐19; and affiliation with an academic or non‐academic healthcare institution in Saudi Arabia. The final voting panel comprised nine experts: five specialists in hematology or HSCT, two in oncology, and two in infectious diseases or transplant infectious diseases. The panel included experts from institutions in Riyadh (*n* = 5), Jeddah (*n* = 2), Makkah (*n* = 1), and Jazan (*n* = 1). All panelists completed conflict‐of‐interest declarations before participation.

### 2.2 Literature Review and Statements Development

A comprehensive literature review was conducted to identify relevant studies, guidelines, and expert opinions on the prophylaxis of SARS‐CoV‐2 infection in patients with HMs. The search strategy included queries for electronic databases (e.g., PubMed, Embase, and the Cochrane Library) and manual searches for reference lists from retrieved articles and relevant guidelines. Various combinations of the following keywords were used to identify potentially eligible literature (“COVID‐19” OR “SARS‐CoV‐2”) AND (“Hematological Malignancies” OR “Hematologic Neoplasms” OR specific malignancy names) AND (“Prophylaxis” OR “Prevention” OR “Vaccination” OR “Tixagevimab” OR “Cilgavimab” OR “Long‐Acting Antibodies” OR “Immunosuppression”). The literature search was limited to articles published in English, with no restriction on publication date. In the case of original publications, only studies with Level 1 quality of evidence, as classified by Wright et al. [18] were considered.

Based on the findings from the literature review, a questionnaire of proposed statements was developed, covering key aspects of prophylaxis strategies for SARS‐CoV‐2 infection in patients with HMs. The questionnaire included topics such as vaccination, passive immunization, pharmacological interventions, and non‐pharmacological measures.

The initial questionnaire comprised 27 draft statements organized into five domains: general preventive strategies, general vaccination recommendations, special vaccination recommendations, pharmacological prophylaxis, and unmet medical needs. Each statement was accompanied, where applicable, by supporting literature identified during the evidence review.

### 2.3 Delphi Process and Consensus Development

The modified Delphi process comprised three sequential steps: an initial electronic survey, a moderated virtual advisory‐board discussion, and electronic recirculation of the revised statements for final voting. In the first step, 27 draft statements were distributed electronically to the nine‐member voting panel. Panelists assessed each statement using the response options “agree,” “disagree,” or “abstain” and could provide free‐text comments and suggestions. Responses were summarized descriptively as the number and percentage of panelists selecting each option. Agreement was calculated as the proportion of “agree” responses among all submitted responses, including abstentions in the denominator. Consensus was predefined as an agreement level of at least 80%. Eleven statements reached consensus in the initial electronic round. Statements that did not meet the consensus threshold, or for which comments raised concerns regarding clarity, feasibility, evidence, redundancy, or terminology, were carried forward for discussion. During the advisory‐board discussion, four statements were removed, one new statement was added, and the remaining statements were revised. In the final step, the revised statements were recirculated electronically to the nine‐member panel for voting. The final set comprised 24 statements, of which 23 achieved 100% agreement, and one achieved 88.9% agreement. The retained records confirm nine electronic voters but do not provide a separate attendance count for the advisory‐board discussion; therefore, a meeting‐specific attendance rate was not estimated. All authors reviewed and approved the final consensus statements.

Voting was anonymous and was conducted electronically using an online survey platform. Experts independently completed the survey and selected either “agree,” “disagree,” or “abstain,” in addition to providing optional comments. Results were aggregated before presentation to the advisory‐board discussion to minimize the influence of dominant panel members during the initial voting phase. Before the advisory‐board discussion, panelists were provided with a summary of the first‐round voting results, including the proportion of agreement for each statement and anonymized comments submitted by participants. This feedback informed the subsequent discussion and revision of statements.

During the moderated virtual advisory‐board discussion, the first‐round voting results were reviewed statement by statement. Panelists discussed statements that did not achieve consensus as well as statements requiring clarification despite reaching the quantitative threshold. Based on these discussions, statements could be retained unchanged, rephrased, reordered, consolidated, added, or removed. Examples of substantive revisions included clarification of vaccine terminology; removal of year‐ and brand‐specific vaccine wording; revision of the granulocyte colony‐stimulating factor recommendation; removal of routine antibody‐titer testing; addition of periodic reassessment for vaccination; reordering of the eligibility groups for mAbs prophylaxis; and qualification of mAbs use according to activity against circulating variants. Two proposed statements in the unmet‐medical‐needs domain were removed after discussion.

Following the advisory‐board discussion, the revised statements were recirculated electronically to the full panel for final voting. The final set comprised 24 consensus statements. Twenty‐three statements received agreement from 9 of 9 panelists, corresponding to 100% agreement, and one statement received agreement from 8 of 9 panelists, corresponding to 88.9%, with one abstention. The exact final result for each statement is reported as n/N and percentage in Tables 1 and 2.

### 2.4 Evidence Assessment and Development of Recommendations

Recommendations were formulated by integrating the available scientific evidence with expert clinical judgment through the modified Delphi process. Evidence quality was categorized according to study design and methodological rigor and is reported for each statement in Tables 1 and 2. In addition to evidence quality, the panel considered the consistency of findings across studies, concordance with international guidance, clinical relevance, feasibility of implementation, and the potential benefits and risks for patients with hematological malignancies. Recommendations supported by randomized controlled trials, systematic reviews, meta‐analyses, prospective studies, or established international guidelines were generally considered more robust. In contrast, recommendations supported primarily by indirect evidence, observational studies, emerging data, or expert opinion were retained when the panel considered them clinically important and necessary for patient care despite the limited evidence base. Consequently, the final wording of each recommendation reflects both the underlying evidence and the degree of consensus achieved among panel members rather than evidence quality alone.

## 3 Results

In the first round, a total of 27 proposed statements were presented to the expert panel for voting. Of them, 11 statements reached a consensus level. After the expert panel meeting, four statements were removed, one new statement was added, and the remaining statements were refined and voted upon in subsequent rounds. The final set of statements consisted of 24 recommendations for the prophylaxis of SARS‐CoV‐2 infection in adults with HMs in Saudi Arabia.

Consensus was reached on four statements concerning the role of general non‐pharmaceutical measures in patients with HMs (Table 1). The panel recommended non‐pharmaceutical measures, including physical distancing, mask use, and hand hygiene, particularly during periods of heightened community transmission. The currently recommended COVID‐19 vaccination was also recommended for household members and caregivers of patients with HMs, as well as for healthcare personnel and unit staff in close contact with these patients. The panel further recommended an individualized assessment of the risks and benefits of granulocyte‐colony stimulating factor administration in neutropenic patients with cancer and COVID‐19.

**TABLE 1:** Consensus statements regarding the role of general preventive strategies and vaccinations in patients with hematological malignancies.

No.	Statement	Consensus level	Certainty | Type/source of evidence	Data origin
General preventive strategies				
1	General non‐pharmaceutical measures are strongly recommended for patients with hematological malignancies, particularly during times of heightened community transmission, including social distancing, mask‐wearing, and hand hygiene.	100%	Moderate | Systematic review/meta‐analysis of observational studies; expert consensus	[19, 20, 21]
2	Alongside general non‐pharmaceutical measures, caregivers and all household members of patients with hematological malignancies should be fully vaccinated with the updated vaccine to mitigate the risk of SARS‐CoV‐2 transmissions.	100%	Low to moderate | Observational study	[22]
3	In the clinical care of patients with hematological malignancies, full vaccination and strict adherence to non‐pharmaceutical protocols are strongly recommended for healthcare personnel and unit staff who are in close contact with the patients.	100%	Low to moderate | Observational study	[22, 23]
4	Weighing the potential risks versus benefits of granulocyte‐colony stimulating factors administration should be considered in neutropenic cancer patients with COVID‐19 because it may lead to worsening clinical outcomes.	100%	Low | Indirect narrative evidence; expert opinion	[24, 25]
II Vaccination				
5	Full vaccination is the recommended first‐line strategy to prevent SARS‐CoV‐2 infection or COVID‐19 complications in patients with hematological malignancies. Vaccination with the updated vaccine should be offered to all patients, including those with previous COVID‐19, transplant recipients, and those with an expected low or very low response rate.	100%	Moderate | Registry and cohort studies; expert guideline	[26, 27, 28]
6	The vaccine choice should follow the national recommendations regarding updated vaccines and availability. Patients with hematological malignancies should receive at least one dose of an updated COVID‐19 vaccine according to the national recommendations.	100%	Moderate | Cohort studies; expert guidelines	[21, 28, 29, 30, 31]
7	Patients with hematological malignancies aged 65 years and older who received one dose of any updated COVID‐19 vaccine should receive one additional dose of an updated COVID‐19 vaccine at least 2 months after the previous updated dose.	100%	Low to moderate | Observational risk study; expert guidance	[32]
8	Vaccination can be administered anytime during the disease course. Patients who undergo any intervention, such as transplantation, chemotherapy, or immunosuppressants, should receive a dose of the updated vaccine at least 2 weeks before the intervention. Patients should receive a second dose after any intervention within the first 3 months and another booster dose after 2 months.	100%	Low to moderate | Prospective and observational immunogenicity studies; expert guidance	[33, 34, 35, 36]
9	Patients with hematological malignancies should be reassessed regularly every 6 months and offered booster doses of the updated COVID‐19 vaccine to ensure optimal protection against SARS‐CoV‐2 and its emerging variants.	100%	Low to moderate | Observational studies	[31, 37]
10	Current data show no safety concerns of COVID‐19 vaccination in non‐transplanted patients. Thus, COVID‐19 vaccination should not delay the treatment of the underlying disease.	100%	Moderate | Observational safety cohorts; expert guidance	[38, 39]
11	Non‐transplant patients who have been vaccinated before or during hematological treatment should be assessed 3–6 months after the end of treatment and revaccinated.	100%	Low to moderate | Expert guidelines informed by systematic evidence review and multidisciplinary consensus	[21, 28]
12	Patients who receive targeted immunotherapy such as anti‐CD20 Monoclonal Antibodies or bispecific monoclonal antibodies should be offered the COVID‐19 vaccine despite the concerns that vaccine‐induced immunity is lower in those patients.	100%	Low to moderate | Observational immunogenicity studies	[33, 35, 40, 41]
13	Patients receiving HSCT or CAR‐T should be fully vaccinated 6 months after treatment, even if such individuals received vaccination before treatment. Early vaccination is recommended for heightened community transmission of SARS‐CoV‐2; later on, every 6 months, patients should be reassessed and consider giving the updated COVID‐19 vaccine.	100%	Moderate | Prospective cohort studies and open‐label trial; expert guidance	[42, 43, 44, 45, 46, 47]

Abbreviations: CAR‐T, chimeric antigen receptor T‐cell therapy; COVID‐19, coronavirus disease 2019; G‐CSF, granulocyte‐colony stimulating factor; HSCT, hematopoietic stem cell transplantation; SARS‐CoV‐2, severe acute respiratory syndrome coronavirus 2.

Nine statements regarding the role of vaccination in patients with HMs reached consensus level. The panel recommended the currently recommended COVID‐19 vaccination as the frontline strategy to prevent SARS‐CoV‐2 infection and severe COVID‐19 outcomes in patients with HMs, regardless of prior COVID‐19, transplant status, or anticipated immune response. Patients should receive at least one dose of an updated COVID‐19 vaccine in accordance with national recommendations and vaccine availability. For patients aged 65 years or older, the panel recommended an additional dose of an updated vaccine at least 2 months after the previous updated dose. All statements regarding the role of vaccination in patients with HMs are illustrated in Table 1.

Eight statements regarding the role of pharmacological prophylaxis in patients with HMs reached a consensus level. The panel recommended pre‐exposure prophylaxis with long‐acting mAbs for immunocompromised patients at heightened risk of severe SARS‐CoV‐2 infection. Priority groups included unvaccinated patients, patients with an inadequate serological response to vaccination, and those on immunosuppressive treatment, lymphoproliferative disease, acute leukemia, or HSCT. The mAbs could be initiated while patients continued their scheduled anticancer treatment. In vaccinated patients, administration was recommended at least 2 weeks after COVID‐19 vaccination. The panel did not recommend high‐titer convalescent plasma (CVP) as post‐exposure prophylaxis in patients with HMs. Additionally, pre‐exposure or post‐exposure prophylaxis with antiviral agents was also not recommended.

Furthermore, three statements were recommended in the consensus regarding the unmet medical needs in patients with HMs. These included the development of a standardized multidisciplinary national protocol for SARS‐CoV‐2 prophylaxis in patients with HMs, improved access to long‐acting mAbs, and timely availability of prophylactic antibodies with activity against circulating SARS‐CoV‐2 variants in Saudi Arabia. All statements regarding the role of pharmacological prophylaxis and unmet medical needs in patients with HMs are illustrated in Table 2.

**TABLE 2:** Consensus statements regarding the role of pharmacological prophylaxis and unmet medical needs in patients with hematological malignancies.

No.	Statement	Consensus level	Evidence strength/design	Data origin
III Pharmacological prophylaxis				
14	Risk stratification is crucial to effectively implementing pre‐exposure prophylaxis, ensuring that individuals at heightened vulnerability are prioritized.	100%	Moderate | Large retrospective and population‐based cohort studies	[48, 49]
15	The use of long‐acting anti‐SARS‐CoV‐2 monoclonal antibodies, as long as they are active against the circulating subvariants in Saudi Arabia, should be considered for high‐risk immunocompromised patients.	100%	High | Phase III randomized controlled trial of pre‐exposure prophylaxis	[9]
16	Pre‐exposure prophylaxis with long‐acting anti‐SARS‐CoV‐2 monoclonal antibodies is recommended, if active against circulating variants, for the following patient groups: Patients who did not receive any dose of the COVID‐19 vaccine. Patients with inadequate serological response (according to the manufacturer's instructions) despite full vaccination. Patients with a high risk of poor vaccine response regardless of the previous vaccination, such as: Patients with lymphoproliferative disease or acute leukemia, particularly those with ongoing anti‐CD20 monoclonal Antibodies therapy or last anti‐CD20 monoclonal Antibodies within 6–12 months Patients with multiple myeloma with ongoing BCMA‐targeted bispecific mAb or last dose within 6–12 months. Those receiving induction chemotherapy for AL. Patients with profound hypogammaglobulinemia (< 4 g/L). Patients with deep lymphopenia (< 500/μL). Patients undergoing HSCT. Patients on higher dose steroids (higher than the physiologic dose for greater than or equal to 2 weeks) for either complication or treatment of primary hematological infections. d Patients with B‐cell aplasia after treatment with CD19+ CAR‐T cells for R/R B‐cell B‐ALL, NHL, and MM.	100%	Low to moderate | Observational risk studies; indirect evidence; expert opinion	[48, 49, 50, 51]
17	Long‐acting monoclonal antibodies should be started once the high‐risk patient is identified, even if the patient is on the scheduled cancer management protocol.	100%	Low | Indirect evidence; expert opinion/panel consensus	Panel consensus; indirect evidence
18	Pre‐exposure prophylaxis with long‐acting monoclonal antibodies should be administered at least 2 weeks after COVID‐19 vaccination. In unvaccinated patients, the vaccine can be administered at any time from the monoclonal antibody dose.	88.9%	Low | Indirect evidence; expert opinion/panel consensus	Panel consensus; indirect evidence
19	Vaccinating donors for allogenic transplants may potentially protect recipients, and it is recommended that donors are vaccinated 2 weeks before donation.	100%	Low | Indirect evidence; expert opinion/panel consensus	Panel consensus; indirect evidence
20	With the available current evidence, high‐titer convalescent plasma as post‐exposure prophylaxis is not recommended for patients with hematological malignancies.	100%	Moderate | Randomized controlled trials in hospitalized patients; indirect evidence for post‐exposure prophylaxis	[52, 53]
21	There is currently no data on the antiviral agents remdesivir, nirmatrelvir/ritonavir, or molnupiravir as pre‐ or post‐exposure prophylaxis in cancer patients, which cannot be recommended.	100%	Low | No direct prophylaxis evidence; expert opinion/panel consensus	Panel consensus; indirect evidence
IV Unmet medical needs				
22	There is a need to develop a standardized, multidisciplinary protocol to guide prophylaxis strategies for SARS‐CoV‐2 infection in patients with hematological malignancies in KSA.	100%	Low | Expert opinion/panel consensus	Panel consensus; indirect evidence
23	Given the observed emergence of novel SARS‐CoV‐2 variants within Saudi Arabia, rapid accessibility to active monoclonal antibodies against most circulating subvariants is imperative to mitigate the risk of COVID‐19 in vulnerable groups.	100%	Low | Indirect evidence; expert opinion/panel consensus	Panel consensus; indirect evidence
24	In the evolving landscape of the COVID‐19 pandemic, it remains critical for the KSA to engage in research targeting emergent variants, ensuring the timely development, validation, and deployment of efficacious therapeutic interventions.	100%	Low | Expert opinion/panel consensus	Panel consensus; indirect evidence

Abbreviations: AL, acute leukemia; BCMA, B cell maturation antigen; CAR‐T, chimeric antigen receptor T; COVID‐19, coronavirus disease 2019; HSCT, hematopoietic stem cell transplantation; LPD, lymphoproliferative disease; SARS‐CoV‐2, severe acute respiratory syndrome coronavirus‐2.

## 4 Discussion

### 4.1 General Preventive Strategies

General non‐pharmaceutical measures, such as social distancing, mask‐wearing, and hand hygiene, are widely recognized as critical strategies to prevent SARS‐CoV‐2 transmission and reduce COVID‐19 risk. Several studies have demonstrated the effectiveness of non‐pharmaceutical measures in reducing the risk of SARS‐CoV‐2 transmission and preventing COVID‐19. Systematic reviews and meta‐analyses found that physical distancing measures were associated with a significant reduction in COVID‐19 incidence [19]. Similarly, a review of 172 observational studies found that mask‐wearing and hand hygiene were associated with a significant reduction in the risk of SARS‐CoV‐2 infection [20]. In addition, in a survey of healthcare professionals working in inpatient oncology and hematology services in Saudi Arabia, 96.4% agreed or strongly agreed that admitted patients should wear a surgical mask during interactions with physicians and nurses [54].

The European Society for Medical Oncology (ESMO)/European Hematology Association (EHA) consensus recommended preventive strategies (such as physical distancing and face masks) for patients with HMs, alongside their relatives and close contacts [21]. The expert panel in this consensus agreed on the importance of non‐pharmaceutical measures for patients with HMs, particularly during times of heightened community transmission (**Statement 1**). Heightened community transmission refers to a situation with a significant increase in COVID‐19 spread. This can occur when SARS‐CoV‐2 circulation is high, and the number of new cases is rapidly increasing.

Alongside general non‐pharmaceutical measures, evidence suggests that the currently recommended COVID‐19 vaccination of household members, caregivers, and healthcare staff can significantly reduce SARS‐CoV‐2 transmission and severe COVID‐19 risk in patients with HMs [22, 23]. Thus, the expert panel recommended that the caregivers and household members of patients with HMs should be vaccinated with the updated vaccine (**Statement 2**). Additionally, the currently recommended COVID‐19 vaccination is strongly recommended for healthcare personnel and unit staff in close contact with patients (**Statement 3**).

Granulocyte‐colony stimulating factors (G‐CSF) are often used to reduce the duration and severity of neutropenia in cancer patients receiving chemotherapy. However, the use of G‐CSF in cancer patients with COVID‐19 is controversial, as it may lead to worsening clinical outcomes. Studies have suggested that G‐CSF administration may be associated with increased inflammation and cytokine release, which can exacerbate COVID‐19‐related lung injury and acute respiratory distress syndrome (ARDS) [24, 25]. Thus, weighing the potential risks versus benefits of G‐CSF administration in neutropenic cancer patients with COVID‐19 is essential (**Statement 4**). The decision to use G‐CSF should be individualized based on the patient's clinical status, neutropenia severity, and the risks of infection and thrombosis.

### 4.2 Vaccination

#### 4.2.1 Impact of Vaccination on Clinical Outcomes

Since the emergence of the COVID‐19 pandemic, patients with HMs have consistently shown higher mortality rates than the non‐cancer population and patients with solid tumors. Previous reports showed that the mortality rate among unvaccinated hospitalized patients with HMs was 15% higher than in the non‐cancer population, with old age and comorbidities as independent risk factors for severe clinical outcomes [55]. However, since the introduction of vaccinations, a cumulative body of evidence has shown a substantial reduction in the rate of severe COVID‐19 infections and related fatalities compared to the pre‐vaccination era. In the EPICOVIDEHA registry, there were significant reductions in COVID‐19‐related mortality rates among patients with HMs during the vaccination era compared to the pre‐vaccination period (12.4% vs. 31%, respectively) [26]. The reduction in mortality was observed among vaccinated patients [26]. An extension analysis of the same registry showed that hospitalization and ICU admission rates among vaccinated patients were lower compared to the pre‐vaccination period [27]. Additionally, local evidence from Saudi Arabia supports vaccination as a key strategy for reducing severe COVID‐19 outcomes. A multicenter study conducted among adults hospitalized with COVID‐19 in the Jazan region during the Omicron wave found that unvaccinated patients had higher risks of mortality, ICU admission, mechanical ventilation, critical illness, and prolonged hospitalization than vaccinated patients [56].

International guidelines like the ESMO/EHA and the European Conference on Infections in Leukemia (ECIL 9) strongly recommended vaccination for patients with HMs [21, 28]. Accordingly, the panel agreed that the currently recommended COVID‐19 vaccination is the frontline prophylactic strategy for SARS‐CoV‐2 prophylaxis or severe COVID‐19 in patients with HMs. The vaccine should be offered regardless of history of previous COVID‐19, transplant status, or expected immune response (**Statement 5**). We also adopt the Centre for Disease Control and Prevention (CDC) recommendations that define vaccination as three doses of mRNA vaccines or two doses of protein subunit vaccine [57]. Vaccine selection (bivalent or updated COVID‐19 vaccine) should follow national recommendations and availability.

#### 4.2.2 Vaccine Immunogenicity in Hematological Malignancies

Current evidence indicates that patients with HMs exhibit lower immunogenicity to COVID‐19 vaccines than healthy populations and those with solid tumors, especially those on immunosuppressive therapies [58]. Patients with chronic lymphocytic leukemia (CLL) and NHL show response rates of 35%–75% and 39%–73%, respectively, with factors such as older age, reduced immunoglobulin levels, lymphopenia, and recent anti‐CD20 or Bruton tyrosine kinase inhibitors (BTKi) treatments contributing to poor responses [29, 40, 59]. Recent data from the international ERIC study further demonstrate that, despite improvements in COVID‐19 outcomes over time, patients with CLL remain at increased risk of severe infection, supporting the need for optimized preventive and vaccination strategies [60]. Patients with multiple myeloma (MM) have a variable response rate of 45%–95%, generally lower than healthy controls, influenced by factors like age and specific treatments [61]. Across all HMs, antibody titers are consistently lower than in healthy controls and decline over time, with lower persistence in actively treated patients [41].

#### 4.2.3 Booster Vaccination

Alongside the low immune response after vaccination in patients with HMs, challenges include the natural waning of responses and the emergence of new variants, leading to breakthrough infection. Current data indicate a higher incidence of breakthrough infections in patients with HMs (15%) compared to the general population. HMs patients with breakthrough infections exhibit a higher risk of morbidity, hospitalization, severe outcomes, and mortality [62, 63, 64].

Thus, a booster vaccination strategy seems reasonable to improve the immune response and minimize the risk of severe disease in patients with HMs. Studies have shown that nearly half of the non‐responders to the initial vaccination series responded after receiving a booster vaccination [65], and a third or fourth dose can increase the antibody titers [31]. Booster doses significantly increased anti‐spike RBD antibodies, including in patients who initially had antibody levels below the positivity threshold. Furthermore, booster shots effectively neutralized titers against SARS‐CoV‐2 variants like Delta and Omicron [30, 37]. It was also found that a fourth dose can restore neutralizing activity against the Wuhan strain to levels achieved after the first booster, even in patients undergoing intensive therapies like anti‐CD38 treatment [66]. Cellular responses are also enhanced with booster doses, with studies showing improved CD4+ T‐cell‐mediated cytokine responses against various strains of SARS‐CoV‐2 [30].

Given the current evidence, several international guidelines recommend booster doses of updated vaccines for patients with HMs [21, 28]. The experts agreed that patients with HMs should receive at least one dose of an updated COVID‐19 vaccine (**statement 6**). As older adults and patients with comorbidities showed an even higher risk of breakthrough infection [32], the panel recommended one additional dose of an updated COVID‐19 vaccine at least 2 months from the last dose in patients with HMs who are aged ≥ 65 years (**Statement 7**).

#### 4.2.4 Special Populations

Although COVID‐19 vaccines can be given anytime during the disease course [21], patients on immunosuppressive treatments (such as anti‐CD38 mAbs) showed weaker humoral responses to SARS‐CoV‐2 variants [33]. Improved humoral responses were observed in patients vaccinated more than 30 days after their last anti‐CD38 infusion [34]. Extending this interval to 3 months from the last treatment dose to vaccination may further boost seroconversion rates [35]. Thus, strategies such as early introduction of vaccination before the period of low immunity were suggested [2]. The panel agreed that the vaccination dose could be introduced at least 2 weeks before any intervention (**Statement 8**). As prior studies showed no difference in initiating vaccination three versus 6 months after transplantation with regard to immunogenicity [36], patients should receive a second dose within the first 3 months and another booster dose after 2 months (**Statement 8**).

The panel also suggested that patients with HMs should be reassessed regularly every 6 months and offered booster doses of the updated COVID‐19 vaccine to ensure optimal protection against SARS‐CoV‐2 and its emerging variants (**Statement 9**).

Data show no specific safety concerns in non‐transplant patients receiving COVID‐19 vaccines compared with the general population. Additionally, evidence suggests no association between vaccination and treatment‐related adverse events [38, 39]. On the other hand, delaying systemic treatments in patients with HMs can adversely affect their prognosis. Thus, the panel agreed that COVID‐19 vaccination should not delay the systemic treatment of HMs (**Statements 10 and 11**) per international guidelines [21, 28]. As elaborated earlier, patients on immunotherapy should receive the currently recommended COVID‐19 vaccination and booster doses despite concerns about lower immunogenicity (**Statement 12**). However, these patients can benefit from additional measures, such as pre‐exposure mAbs, discussed in the next chapter.

Previous studies showed that COVID‐19 vaccination led to a response rate in allogeneic HSCT patients, with earlier vaccination post‐HSCT, lower lymphocyte counts or IgG levels, active graft‐versus‐host disease (GVHD), and ongoing or recently discontinued immunosuppression as risk factors for poor responses [42, 43, 44, 45]. Notably, robust polyfunctional memory T‐cell and serological responses have been observed after the second vaccine dose in patients with chronic GVHD and previous poor serological responses, suggesting repeated vaccinations could enhance responsiveness in this group [46]. Few studies have also shown the effectiveness of a third dose in patients without a previous response and those with a weak prior response [47]. Since vaccinated patients undergoing HSCT likely lose all vaccine‐induced immune memory, they should be vaccinated 6 months after treatment. Patients should be reassessed every 6 months for revaccination (**Statement 13**).

#### 4.2.5 Vaccine Compliance

Improving vaccine compliance among patients with HMs requires personalized counseling that addresses their specific disease, treatment, and concerns about vaccine safety and effectiveness. The treating hematologist or oncologist should provide clear and tailored recommendations, as patients with cancer value advice from their oncology team and prefer information explaining how vaccination may interact with their cancer and treatment. Educational and communication support for healthcare professionals may further facilitate informed discussions and help address vaccine hesitancy [67].

### 4.3 Pharmacological Prophylaxis

#### 4.3.1 Residual Risk Despite Vaccination

Despite vaccination, patients with HMs remain at significant risk of breakthrough COVID‐19 infections, which adversely impact their clinical outcomes. A retrospective cohort study analyzed electronic health records from December 2020 to October 2021 of 514 413 vaccinated patients from 63 healthcare organizations in the United States. Among these, 5956 had HMs. The cumulative incidence of breakthrough COVID‐19 in this cohort was 13.4%, significantly higher than 4.5% in those without malignancies. These patients exhibited a markedly higher risk of hospitalization (37.8%) and mortality (5.7%) compared to those without breakthrough infections (hazard ratio [HR] = 34.49 for hospitalization and HR = 10.25 for mortality) [32].

The INFORM trial, conducted in the United Kingdom (UK), identified approximately 12 million patients aged 12 years and older in January 2022. Of these, around half a million were immunocompromised, with 85% having received at least three vaccine doses. Despite constituting only 3.9% of the population, these patients accounted for a disproportionate 22% of COVID‐related hospitalizations, 28% of ICU admissions, and 24% of deaths. Notably, patients with HMs who had undergone recent treatment exhibited significantly higher risks: hospitalization risk was 3.62 times higher, ICU admission risk was 13.58 times higher, and mortality risk was 3.72 times higher than the general population. For those who had undergone stem cell transplants within the past 2 years, the risks were even more pronounced: hospitalization risk was 10.96 times, ICU admission risk was 24.92 times, and mortality risk was 5.16 times higher [48].

The EPOCH study in the United States (US) reviewed the data of nearly 18 million health plan members. Immunocompromised individuals represented 2.7% of this population but accounted for 23.5% of COVID‐related hospitalizations and 48.7% of ICU admissions. The financial burden was substantial, with total COVID‐19‐related hospitalization costs nearing $1 billion and a mean cost of $64 029 per patient. Immunocompromised patients, including those with HMs, contributed 31% of total COVID‐19 hospitalization costs [49].

Collectively, such data underscore the inadequacy of current vaccination strategies to elicit a robust immune response in these patients, necessitating additional protective measures. The current evidence also suggests a potential impact of the type of HMs and treatment modalities on the outcomes of breakthrough COVID‐19 infections. Patients with CLL and MM were found to have notably high mortality rates. In CLL patients, the COVID‐19 mortality rate is approximately 30%, while it escalates to 54.6% in MM patients. More recent data from the large international European Research Initiative on CLL (ERIC) study suggest that outcomes have improved substantially over the course of the pandemic, likely reflecting the impact of vaccination, antiviral therapies, and improved supportive care. Nevertheless, patients with CLL continue to experience a clinically meaningful risk of severe COVID‐19 and mortality, particularly those of advanced age, with comorbidities, or receiving active treatment. These findings emphasize that despite improvements in outcomes, patients with CLL remain a vulnerable population requiring continued preventive strategies and careful risk stratification [60]. Additionally, therapies that deplete B‐cells, such as anti‐CD20 mAbs, BTKi, and chimeric antigen receptor T‐cell therapy (CAR‐T) treatments, have been associated with poor COVID‐19 outcomes in patients with lymphoma. Patients with acute myeloid leukemia (AML), who often experience severe granulocytopenia, also face an increased risk of severe COVID‐19, particularly if they are undergoing autologous or allogeneic HSCT. Additionally, prolonged SARS‐CoV‐2 infection has been observed in some patients with B‐cell lymphoid malignancies [50, 51].

Thus, the expert agreed that risk stratification is crucial to understanding the risk of breakthrough infections in patients with HMs and assessing the need for pre‐exposure prophylaxis (**Statement 14**).

#### 4.3.2 Long‐Acting mAbs

A crucial measure in those with heightened vulnerability to severe COVID‐19 is the use of pre‐exposure long‐acting mAbs [68]. Intramuscular AZD7442, a combination of two long‐acting mAbs (T/C), was designed for the prevention and treatment of COVID‐19 by targeting the spike protein of SARS‐CoV‐2, preventing the virus from entering and infecting human cells [69]. This dual‐action approach enhances its efficacy in neutralizing the virus and offers a prolonged duration of protection [68].

Evidence supporting the prophylactic use of long‐acting mAbs is derived primarily from pre‐exposure prophylaxis studies. The efficacy of T/C for pre‐exposure prophylaxis was demonstrated in the PROVENT study, which included patients at high risk of insufficient response to vaccination or increased risk of COVID‐19 infection. In this study, COVID‐19 infection occurred in eight patients in the T/C group compared to 17 in the placebo group (*p* < 0.001). Additionally, all severe or critical cases and COVID‐19‐related deaths (five and two cases, respectively) were reported only in the placebo group [9]. Further supporting evidence comes from a retrospective study involving 203 patients with HMs, which found that the pre‐exposure prophylaxis led to a comparable risk of severe COVID‐19 in high‐risk group patients compared to the general risk population [70]. Another real‐world study found that T/C led to a lower risk of SARS‐CoV‐2 infection than receiving only a fourth vaccine dose [71]. Within the Saudi setting, AZD7442 was evaluated at King Saud Medical City in Riyadh as part of EVOLVE, a prospective, multicenter observational study designed to characterize the utilization and clinical outcomes of T/C as pre‐exposure prophylaxis in GCC countries. Although the study has been completed, its clinical outcomes have not been publicly reported, and Saudi‐specific effectiveness data in patients with HMs remain limited [72].

However, as of January 2023, the Food and Drug Administration (FDA) revised the Emergency Use Authorization for T/C due to the prevalence of new Omicron subvariants, which account for over 90% of infections in the United States and are less susceptible to these mAbs [73]. Consequently, sipavibart (formerly AZD3152) was developed to act against the new subvariants of the current COVID‐19 strains. In the SUPERNOVA trial, sipavibart met its primary endpoint in reducing the incidence of symptomatic COVID‐19 compared to T/C and placebo in immunocompromised patients [74].

VYD222 is another long‐acting mAb that provides broad activity against several SARS‐CoV‐2 variants, such as XBB.1.5. Single‐dose VYD222 has demonstrated a well‐tolerated safety profile in healthy adults [75], which led to investigating its efficacy in preventing severe COVID‐19 among immunocompromised patients, including patients with HMs, in the Phase III CANOPY trial. Among those with solid tumors or HMs, VYD222 was associated with no incidence of symptomatic COVID‐19 and high titers of serum virus‐neutralizing antibodies against SARS‐CoV‐2 variants on day 28. VYD222 was also well‐tolerated, with no incidence of serious adverse events [10].

Regeneron's REGN17092 is another potential prophylactic mAb, which is characterized by preserved efficacy due to the high conservation rate of the targeted epitope [11]. Currently, a randomized, placebo‐controlled trial is ongoing to understand the safety profile of different doses of REGN17092 (NCT05923424). Likewise, the anti‐receptor‐binding protein (RBD) AER‐800, which combines AER003/P4J15 and AER004, is currently under investigation for its prophylactic potential in immunocompromised patients [12].

Long‐acting anti‐SARS‐CoV‐2 mAbs have provided proof of concept for passive pre‐exposure prophylaxis in individuals unlikely to develop an adequate vaccine response. However, their clinical effectiveness is intrinsically variant‐dependent because viral evolution, particularly changes in the spike protein, can reduce or eliminate neutralizing activity. Consequently, evidence for mAbs must be interpreted in relation to the variants circulating during the study and should not automatically be extrapolated to later variant periods [12, 76].

Current recommendations should therefore be agent‐specific, variant‐specific, jurisdiction‐specific, and time‐sensitive. mAbs should be considered only when recent neutralization or susceptibility evidence demonstrates activity against circulating variants, the product has approval or authorization from the relevant local regulator, and it is locally available. Eligibility based on immune compromise identifies patients who may benefit from passive immunoprophylaxis, but it does not establish that any particular antibody remains active. Where contemporaneous Saudi susceptibility data are unavailable, international surveillance and product‐specific regulatory information may inform the assessment, but local approval and availability remain necessary [13, 76].

Within this framework, the panel agreed that pre‐exposure long‐acting mAbs should be used for immunocompromised patients with heightened vulnerability to severe SARS‐CoV‐2 infection (**Statement 15**), provided that the agent has demonstrated activity against circulating variants and is locally approved and available. The panel identified several high‐risk groups, including unvaccinated patients, patients with inadequate serological response to vaccination, patients on immunosuppressive treatment, lymphoproliferative diseases or AML, and patients undergoing HSCT, among others (**Statement 16**). The mAbs should be started, even if the patient is under treatment. In the case of vaccinated patients, the long‐acting mAbs should be administered at least 2 weeks after vaccination (**Statements 17 and 18**). The characteristics, variant coverage, and available clinical evidence for the long‐acting mAbs discussed above are summarized in Table 3.

**TABLE 3:** Summary of mAbs for SARS‐CoV‐2 prophylaxis.

Agent	Mechanism	Variant coverage	Trial/clinical data
Tixagevimab/cilgavimab (AZD7442)	Two long‐acting anti‐spike antibodies that block viral entry	Activity reduced against dominant Omicron subvariants cited for January 2023	PROVENT Phase III RCT; retrospective and real‐world HMs studies
Sipavibart (AZD3152)	Long‐acting neutralizing antibody developed for newer variants	Developed for newer circulating subvariants; exact contemporaneous Saudi coverage requires surveillance	SUPERNOVA Phase III met its reported primary prevention endpoint
VYD222 (pemivibart)	Extended‐half‐life IgG1 neutralizing antibody	Reported activity against variants including XBB.1.5	Phase I safety; Phase III CANOPY immunocompromised subgroup
REGN17092	Neutralizing antibody targeting a conserved epitope	Expected breadth based on epitope conservation; clinical coverage not yet established	Randomized placebo‐controlled dose‐ranging trial NCT05923424
AER‐800	Combination AER003/P4J15 plus AER004 targeting the receptor‐binding domain	Coverage not established in the cited manuscript evidence	Preclinical/early clinical pipeline review

#### 4.3.3 Other Pharmacological Approaches

Although initial single‐arm studies suggested that convalescent plasma (CVP) may reduce the risk of COVID‐19 complications [52, 53], more recent studies showed no impact of CVP on the recovery or mortality of patients with severe COVID‐19 [77, 78]. Thus, post‐exposure CVP is not recommended for patients with HMs (**Statement 20**). As the current data do not show significant benefits of antiviral treatment (such as remdesivir or nirmatrelvir/ritonavir) on the incidence and severity of symptomatic COVID‐19 [50]. Pre‐or post‐exposure prophylaxis with antiviral agents is not recommended (**Statement 21**).

### 4.4 Unmet Medical Needs

Although considerable progress has been made in developing COVID‐19 prophylaxis strategies and therapeutic interventions for patients with HMs, several unmet needs remain. First, there is a need to develop a standardized, multidisciplinary protocol to guide prophylaxis strategies for SARS‐CoV‐2 infection in patients with HMs in Saudi Arabia. The protocol should consider the unique characteristics of this patient population, including their immunocompromised status and the potential impact of their underlying disease and treatments on vaccine efficacy and safety. The protocol should also provide clear guidance on non‐pharmaceutical interventions and the role of caregivers and household members in preventing transmission.

Second, given the observed emergence of novel SARS‐CoV‐2 variants within Saudi Arabia, rapid accessibility to active mAbs against most circulating subvariants is imperative to mitigate the risk of COVID‐19 in vulnerable groups. mAbs have shown promise in preventing and treating COVID‐19, particularly in high‐risk populations. However, their effectiveness may be compromised by the emergence of new variants. Therefore, ongoing surveillance of circulating variants and timely evaluation of susceptibility data are essential to ensure that prophylactic recommendations remain clinically relevant.

Finally, in the evolving landscape of the COVID‐19 pandemic, it remains critical for Saudi Arabia to engage in research targeting emergent variants, ensuring the timely development, validation, and deployment of efficacious therapeutic interventions. This includes not only mAbs but also antiviral therapies, immunomodulatory agents, and other novel treatments. Collaboration between academic institutions, government agencies, and industry partners will be essential to drive innovation and improve outcomes for patients with HMs and other high‐risk populations.

### 4.5 Study Limitations

This consensus has several limitations. The modified Delphi panel included nine experts selected by convenience sampling, which may limit representativeness. Most recommendations rely on observational studies, immunogenicity endpoints, international guidance, or expert opinion rather than trials conducted in Saudi patients with HMs. In addition, the SARS‐CoV‐2 variant landscape continues to evolve rapidly, and the activity of mAbs may change substantially over time as new variants emerge. Consequently, recommendations regarding passive immunoprophylaxis may require periodic reassessment as new susceptibility, effectiveness, and regulatory data become available. Some recommendations, particularly those concerning pharmacological prophylaxis and unmet medical needs, were based primarily on expert consensus because high‐quality disease‐specific evidence was unavailable. These statements may therefore be interpreted as consensus‐based clinical guidance reflecting current expert practice rather than definitive evidence‐based recommendations. Furthermore, Saudi‐specific effectiveness data, especially regarding long‐acting mAbs, remain limited, necessitating extrapolation from international studies. Finally, although the educational grant sponsor had no role in the study design, evidence review, consensus development, voting process, interpretation of findings, manuscript preparation, or publication decision, the possibility of a perceived influence related to industry funding cannot be completely excluded.

## 5 Conclusion and Future Directions

The consensus outlined in this document underscores the critical need for tailored prophylactic strategies to protect high‐risk immunocompromised patients from COVID‐19, particularly within the context of HMs. Key recommendations include the need for booster doses of the updated vaccines, according to national availability, and the use of long‐acting mAbs for pre‐exposure prophylaxis when activity against circulating SARS‐CoV‐2 variants has been demonstrated and the agent is locally available and approved. Ongoing surveillance of emerging variants remains essential to maintain the clinical relevance of mAbs‐based prevention strategies. Effective risk stratification is essential to prioritize and protect high‐risk patients with HMs. Developing standardized, multidisciplinary protocols for prophylaxis against SARS‐CoV‐2 in patients with HMs is imperative. Continued research and adaptation to the evolving pandemic landscape will help Saudi Arabia safeguard vulnerable populations, mitigate the impact of COVID‐19, and improve patient outcomes.

## Author Contributions

**Mubarak Al‐Mansour:** conceptualization, project administration, writing – original draft, methodology, investigation. **Ayman Alhejazi:** investigation, methodology, writing – original draft. **Manal Ahmed Alsaggaf:** methodology, investigation, writing – original draft. **Hafiz Mosa Malhan:** methodology, investigation, writing – original draft. **Riad El Fakih:** methodology, investigation, writing – original draft. **Reyad Dada:** methodology, investigation, writing – review and editing. **Ghazi Saud Alotaibi:** methodology, investigation, writing – review and editing. **Reem S. Almaghrabi:** methodology, investigation, writing – review and editing. **Majid Alshamrani:** methodology, investigation, writing – review and editing.

## Funding

This work was supported by an unrestricted educational grant from AstraZeneca UK Ltd. The sponsor had no role in study conception or design, selection or review of the evidence, development or administration of the modified Delphi process, formulation or voting on the consensus statements, interpretation of the findings, preparation or approval of the manuscript, or the decision to submit the manuscript for publication. Responsibility for the content and the decision to publish remained solely with the authors.

## Ethics Statement

The authors have nothing to report.

## Consent

The authors have nothing to report.

## Conflicts of Interest

The authors declare no other conflicts of interest relevant to this work. The manuscript discusses monoclonal antibodies developed or marketed by AstraZeneca as well as products from other manufacturers.

## Data Availability

Data sharing is not applicable to this article as no datasets were generated or analyzed during the current study.
